# Digital Interventions in Neuro‐Rehabilitation: Gotcha! trial of an app‐based therapy for proper name anomia in people with dementia

**DOI:** 10.1002/alz70856_097154

**Published:** 2025-12-24

**Authors:** Aygun Badalova

**Affiliations:** ^1^ University College London, London, United Kingdom, United Kingdom; Institute Of Neurology, London, United Kingdom, United Kingdom

## Abstract

**Background:**

Proper name anomia is a common experience that can become unpleasantly amplified in people with dementia (PWD). The Gotcha! app aims to provide practice‐based therapy for PWD enabling them to spontaneously retrieve the names of key people in their lives.

**Methods:**

Gotcha! is a digital confrontation naming therapy app. PWD supply images and names of the people they want to be able to name and train on one face per day for six weeks. We employed a single‐case experimental design with weekly testing of free‐naming in both six‐week blocks (pre therapy and during therapy). A novel speech verifier was used to provide real‐time feedback (Barbera et al. 2020). PWD also had an MEG scan before and after the therapy block where they attempt to name pictures of familiar (trained) and famous (untrained) faces. We interrogated the behavioural data in two ways: 1) a within‐subject non‐parametric analysis using Tau‐U metric (Parker et al. 2011); 2) a parametric group analysis using an ANOVA. MEG data were analysed in SPM. We measured source localised gamma‐band (30‐80 Hz) power 0‐1000 ms after the onset of a face. We ran a group‐based 2x2 factorial analysis on the resultant images (familiar vs. famous; pre‐ vs. post‐therapy) using a repeated‐measures ANOVA to look for changes in power.

**Result:**

The trial is ongoing (target = 45 PWD). Results from the first 20 PWD to complete the trial demonstrate:

1) Tau‐U. 80% showed a positive trend with better naming during the training phase with 8/20 reaching statistical significance.

2) ANOVA showed a significant effect at the group level of training>baseline phase, F (1,19)=13.18, *p* = 0.01

Results from the MEG analysis of 14 PWD: We identified a large cluster of 813 voxels situated in the left ventral temporal lobe (MNI: ‐50 ‐28 ‐26, *F* = 9.19, *p* = 0.004) where gamma reduction was associated with training (pre‐post) of familiar faces, but not (untrained) famous faces.

**Conclusion:**

Gotcha! app‐based therapy for proper name anomia works for the majority of PWD in our trial thus far. This is the first study to demonstrate that the left ventral temporal lobe region supports practice‐based retrieval of familiar face‐name associations in PWD.

